# *Porphyromonas gingivalis*-mediated disruption in spiral artery remodeling is associated with altered uterine NK cell populations and dysregulated IL-18 and Htra1

**DOI:** 10.1038/s41598-022-19239-9

**Published:** 2022-08-30

**Authors:** Tanvi Tavarna, Bryce Wolfe, Xiao-jun Wu, Leticia Reyes

**Affiliations:** Department of Pathobiological Sciences, School of Veterinary Medicine, University of Wisconsin - Madison, 2015 Linden Drive, Madison, WI 53706 USA

**Keywords:** Microbiology, Diseases, Pathogenesis

## Abstract

Impaired spiral artery remodeling (IRSA) underpins the great obstetrical syndromes. We previously demonstrated that intrauterine infection with the periodontal pathogen, *Porphyromonas gingivalis,* induces IRSA in rats. Since our previous studies only examined the end stage of arterial remodeling, the aim of this study was to identify the impact of *P. gingivalis* infection on the earlier stages of remodeling. Gestation day (GD) 11 specimens, a transition point between trophoblast-independent remodeling and the start of extravillous trophoblast invasion, were compared to late stage GD18 tissues. *P. gingivalis* was found in decidual stroma of GD11 specimens that already had reduced spiral artery remodeling defined as smaller arterial lumen size, increased retention of vascular smooth muscle, and decreased invasion by extravillous trophoblasts. At GD11, *P. gingivalis*-induced IRSA coincided with altered uterine natural killer (uNK) cell populations, decreased placental bed expression of interleukin-18 (IL-18) with increased production of temperature requirement A1 (Htra1), a marker of oxidative stress. By GD18, placental bed IL-18 and Htra1 levels, and uNK cell numbers were equivalent in control and infected groups. However, infected GD18 placental bed specimens had decreased TNF + T cells. These results suggest disturbances in placental bed decidual stroma and uNK cells are involved in *P. gingivalis*-mediated IRSA.

## Introduction

Impaired spiral artery remodeling (IRSA) is associated with an array of pregnancy complications including recurrent early pregnancy loss, early-onset preeclampsia with and without fetal growth restriction, preterm labor, preterm premature rupture of membranes, late spontaneous abortion, and abruptio placentae^1–3^. *Porphyromonas gingivalis,* a human periodontal pathogen, is implicated early pregnancy loss, early-onset preeclampsia with and without fetal growth restriction, preterm labor, and preterm premature rupture of membranes^4–9^. We postulated that this microbe promotes pregnancy complications by disrupting spiral artery remodeling during pregnancy. Using a rat model of periodontitis, we previously established that intrauterine infection with certain wildtype strains *P. gingivalis* induce IRSA^10,11^. Because our previous work only examined spiral artery remodeling in late stage pregnancy, the objective of this study was to identify the processes of spiral artery remodeling affected by *P. gingivalis*.

Physiologic remodeling of human uterine spiral arteries is a complex temporal process that involves cooperation between various cell populations within the placental bed. During the “extravillous trophoblast-independent” (EVT) phase, decidualized stromal cells, uterine NK (uNK) cells, and decidual macrophages (MΦ) contribute to the breakdown of the spiral arterial wall, which includes vascular smooth muscle cell (VSMC) dedifferentiation and migration away from the vessel wall along with breakdown of the vessel extracellular matrix^12–14^. As pregnancy progresses and uNK cell populations disappear from the decidua, EVT that invade the decidual stroma (interstitial EVT) or vessel lumen (endovascular EVT) continue remodeling the spiral arteries, which is often referred to as the “trophoblast-dependent phase^15^. A fully remodeled spiral artery has a large lumen diameter, and the arterial wall is replaced with EVT intermixed with a fibrinoid matrix^16^. Any disturbance or dysregulation during the various phases of spiral artery remodeling can produce IRSA^17^.

In this study we examined gestation day (GD) 11 specimens in which the myometrium is decidualized^18^ and is in transition between the trophoblast-independent phase of uterine spiral arterial remodeling and the start of trophoblast invasion. We found that by gestation day (GD) 11, *P. gingivalis*-infected specimens displayed reduced spiral artery remodeling based on smaller spiral arterial lumen size, increased retention of vascular smooth muscle, and decreased invasion by endovascular invasive trophoblast cells. *P. gingivalis*-induced IRSA at GD11 also exhibited changes in factors implicated in spiral artery remodeling such as altered uterine natural killer (uNK) cell populations, decreased placental bed expression of interleukin-18 (IL-18), and increased production of temperature requirement A1 (Htra1). By GD18, infection-induced changes in placental bed IL-18, Htra1, and uNK cells were equivalent between control and infected groups. However, there was a reduction in TNF + T cells around the spiral arteries in infected GD18 specimens.

## Results

### *P. gingivalis*-mediated disruption of spiral artery remodeling is present by GD11

We previously demonstrated that by GD18, *P. gingivalis* infection disrupts the physiologic remodeling of uterine spiral arteries characterized by reduced arterial lumen size, increased retention of spiral VSMC, and decreased extravillous trophoblast in the placental bed^11^. The objective of this study was to determine if infection affected the “trophoblast-independent” phase of vascular remodeling. We chose GD11 as our study timepoint since the mesometrial triangle is decidualized at this time^18^ but extravillous trophoblasts should not have invaded the mesometrium^19^.

**Figure 1:**
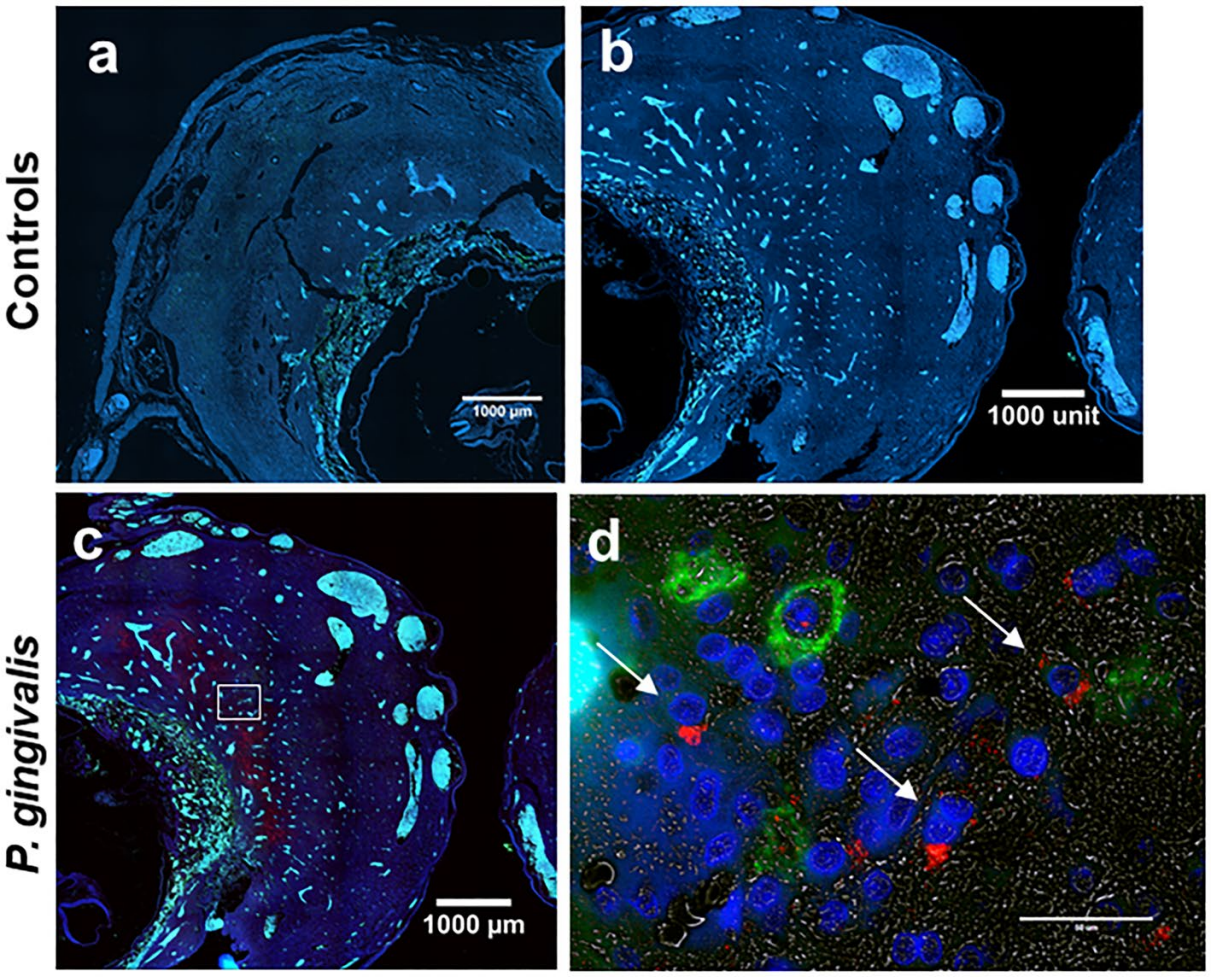
Representative images of *P. gingivalis* in GD11 specimens. (**a**) Stained section from an uninfected control. (**b**) Corresponding isotype control for *P. gingivalis* positive specimen. (**c**) Low magnified image of a *P. gingivalis* positive specimen. Boxed region indicates the region that is pictured in (**d**). Transillumination (grey) was used to define the tissue architecture in decidual stroma in (**d**). Scale bar = 50 µm. (**a–c**) Are tiled composites of individual pictures taken at × 10 magnification with an EVOS Auto FL imaging system. Scale bar = 1000 µm. Tissue sections were dual stained with an antibody to NK cell marker, a-natural killer cell activation structures (green) and *P. gingivalis* (red, white arrows). DAPI (blue) was used as a nuclear stain.

Immunofluorescent staining was used to confirm *P. gingivalis* infection of the uteroplacenta (Fig. 1). *P. gingivalis* antigen was not detected in control specimens. *P. gingivalis* was mostly detected in the decidual stroma near the maternal–fetal interface. In some specimens, bacteria were detected in the periphery of the placental bed surrounding spiral arteries.

**Figure 2:**
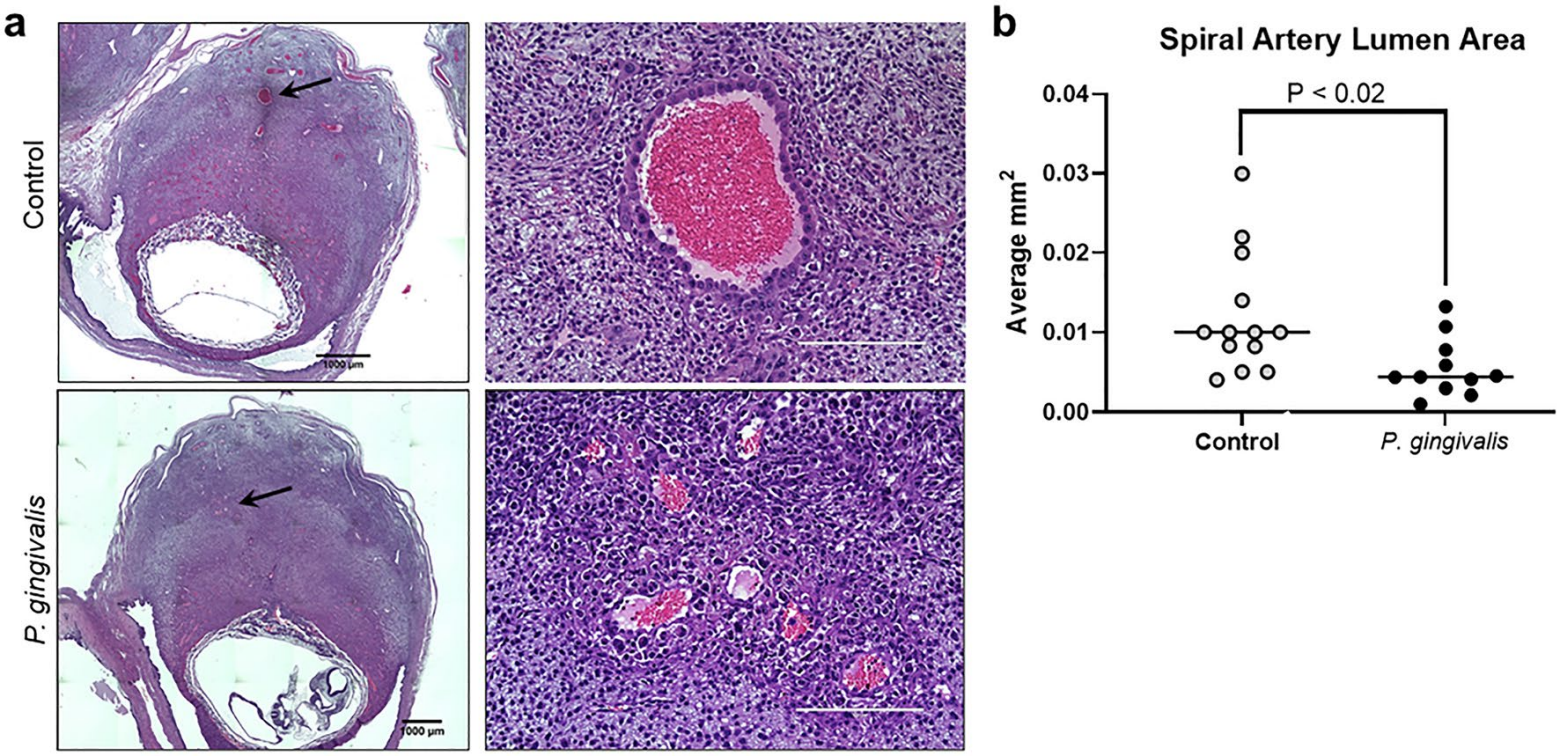
Impact of *P. gingivalis* infection on GD11 spiral artery lumen size. (**a**) Representative images from control and *P. gingivalis*-infected specimens. Left panels are tiled composites of individual pictures taken at × 10 magnification with an EVOS Auto FL imaging system. Scale bar = 1000 µm. Black arrows mark the spiral artery segments pictured in the corresponding right panels. White scale bar = 200 µm. (**b**) Spiral artery lumen area measurements from control and *P. gingivalis*-infected specimens. Horizontal lines indicate the mean ± SD. Data was analyzed by student’s t test.

**Figure 3:**
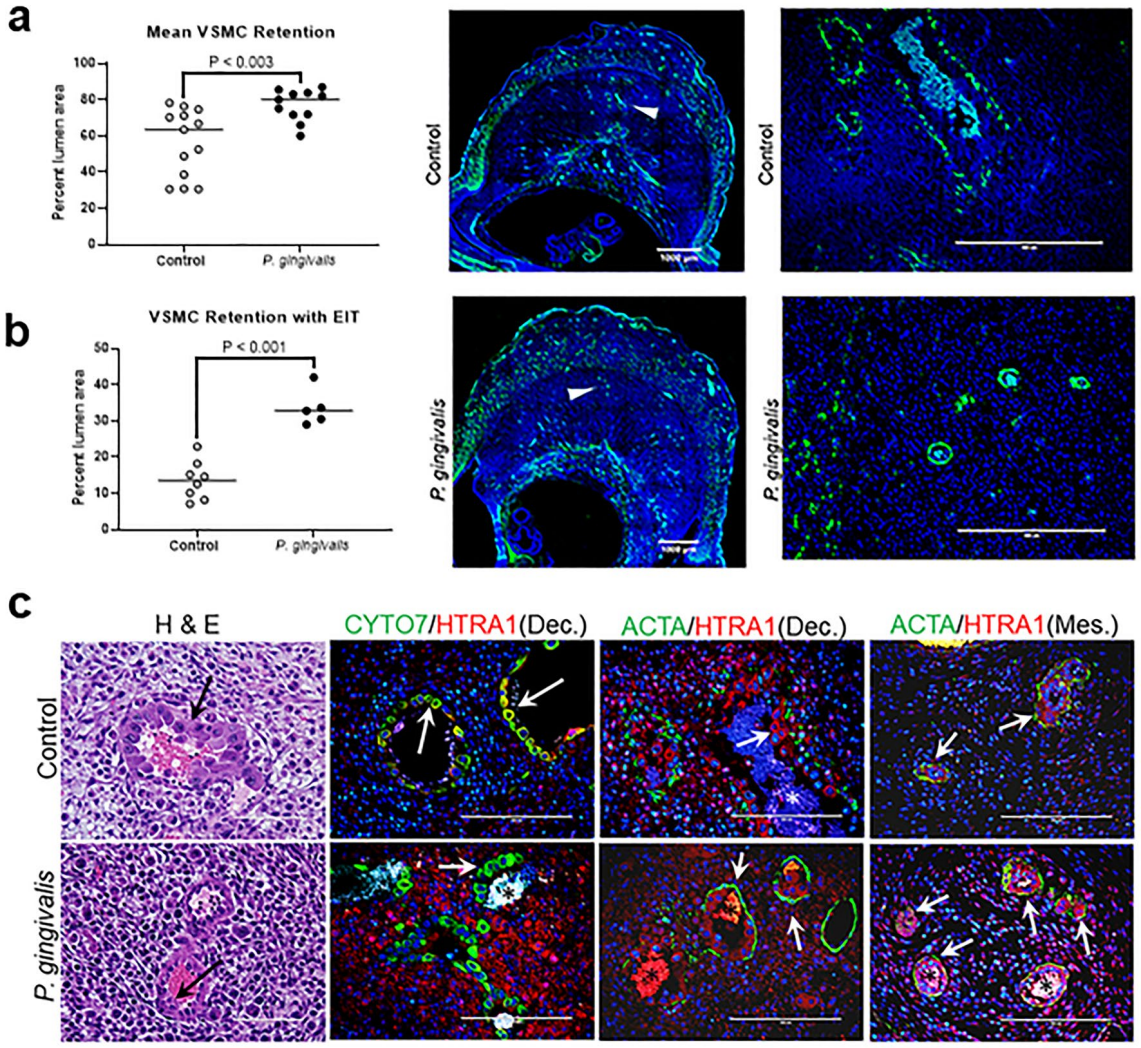
Retention of spiral arterial VSMC and invasive endovascular trophoblasts in GD11 specimens. (**a**) Mean percent circumferential area of spiral arterial VSMCs in specimens with and without endovascular invasive trophoblast (EIT). Each data point represents the average value of all area measurements taken within the middle portion of the utero-placenta. Horizontal bars are mean ± SD. Representative images of spiral arterial VSMC detected by staining for smooth muscle actin (ACTA, green) and DAPI staining for nuclei. Left panels are tiled composites of individual pictures taken at × 10 magnification with an EVOS Auto FL imaging system. Scale bar = 1000 µm. White arrowheads mark the spiral artery segments pictured in the corresponding right panels. White scale bar = 200 µm. (**b**) Mean percent circumferential area of VSMC in spiral arterial loops containing EIT. Horizontal lines = mean ± SD. (**c**) Representative images of EIT in hematoxylin and eosin stained sections (H&E), and by immunostaining with an antibody to cytokeratin 7(CYTO7, green) and high temperature requirement A1 (Htra1, red). Graphical data was analyzed by student’s t test.

The degree of spiral artery remodeling was assessed by dilation of the arterial lumen, loss of the vascular smooth muscle cell (VSMC) layer, and invasion of the uterine tissue by extravillous trophoblasts^20^. Analysis was performed on sections that included the center of the placental bed where vascular remodeling is most consistent (Fig. 2a)^21^. Spiral arterial lumen size was significantly reduced in the infected group compared to controls (Fig. 2b, P < 0.02). Infected animals also had a greater proportion of retained spiral arterial VSMC compared to controls (Fig. 3a,b, P < 0.003).

In contrast to a previous study^19^, endovascular invasive trophoblasts were present in a subset of control and infected specimens. However, the proportion of infected specimens positive for endovascular invasive trophoblasts was significantly less than controls; 10 of 34 (29%) in the decidua of infected animals vs. 27 of 40 (68%) in the control group (P ≤ 0.002 by Fisher’s exact test). In specimens in which endovascular invasive trophoblasts reached the mesometrium: 1 of 34 (3%) in the infected group vs. 11 of 40 (28%) in the control group (P ≤ 0.004 by Fisher’s exact test).

Since endovascular invasive trophoblasts participate in the breakdown of the arterial wall and removal of spiral arterial VSMC^19,22^, VSMC density in endovascular invasive trophoblast positive arterial segments was reanalyzed. Despite the presence of endovascular invasive trophoblasts, the proportion of retained VSMC was greater in infected animals than in controls (Fig. 3b, P < 0.002). Interstitial invasive trophoblasts were not detected in any GD11 specimens (Fig. 3), which is consistent with previous reports^23^.

Retained VSMC in control vs infected groups were also qualitatively different (Fig. 3c). In control specimens the spiral arterial wall in the decidua was mostly or completely obliterated with few to no VSMC present. Any remaining VSMC within the decidua were largely dedifferentiated (round shape with less smooth muscle actin). In the mesometrial triangle, the spiral arteries in control specimens showed partial disorganization of the arterial wall and VSMC showed varying degrees of rounding and decreased smooth muscle actin. In infected animals, decidual arterial segments were less disorganized and remaining VSMC retained contractile features (fusiform shape and increased smooth muscle actin). In the mesometrial triangle, the spiral arteries in infected specimens retained their structure and VSMC contractile features. Thus, despite the presence of endovascular invasive trophoblasts at GD11, spiral arterial remodeling was significantly reduced in *P. gingivalis* infected specimens.

### Vascular necrosis with thrombosis was more prevalent in *P. gingivalis*-infected specimens

Hematoxylin and eosin stained GD11 specimens were examined for coagulative necrosis and perivascular necrosis with or without thrombosis since these lesions have been observed in GD18 placental bed tissues^10,11^. Coagulative necrosis with or without hemorrhage was present at the decidual-placental junction in 6 of 34 (18%) control specimens and 2 of 40 (5%) infected specimens (P = 0.1322 by Fisher’s exact test, representative images available in Supplement File Fig. S1a). In contrast, decidual vascular necrosis with thrombosis of affected vessels was 8 of 40 (20%) infected specimens whereas only 1 of 34 (3%) of controls had this lesion (Fig. S1b, P ≤ 0.0332 by Fisher’s exact test).

### Infection and gestational stage effects on maternal serum cytokine/chemokine concentrations

In our previous study, we found that *P. gingivalis* infection did not alter circulating cytokines and chemokines in GD18 dams^11^. This contradicted previous studies that reported experimental infection with *P. gingivalis* induced TNF and/or IL-1 expression in pregnant animals^24,25^. To determine if maternal systemic inflammation was increased at GD11, cytokines and chemokines in maternal serum were profiled using the same methodology for GD18 dams^11^. To be able to compare GD11 to GD18 sera, both groups were analyzed within the same assay. GM-CSF was not detected in any of the specimens. Overall, infection did not alter maternal cytokine/chemokine profiles at GD11 (Fig. S2a, Supplement File). To determine if gestational stage had any effect on circulating maternal cytokine/chemokine concentrations, GD11 groups were compared to GD18. There were no differences in maternal serum cytokine/chemokine concentrations between control GD11 and GD18 groups (Fig. S2b, Supplement File). Among infected groups, GD11 dams had higher circulating IL-17A (P < 0.02), MIP-2 (P < 0.02) and IL-12p70 (P < 0.03) compared to GD18 dams (Fig. S2c, Supplement File).

### Lymphocyte populations are altered in *P. gingivalis* infected dams

Placental bed leukocytes, particularly uterine NK cells (uNK) and macrophages (MΦ), play important roles in spiral artery remodeling^13,26,27^. Therefore, flow cytometry was used to measure placental bed uNK cell (CD45^+^/CD161a^+^/CD3^−^/CD68^−^), MΦ (CD45^+^/CD68^+^/CD3^−^) populations in both GD11 and GD18 specimens. T cells were identified as (CD45^+^/CD3^+^/CD68^−^) since they also express CD161a. Gating strategy is available in Supplement File, Fig. S3a.

**Figure 4:**
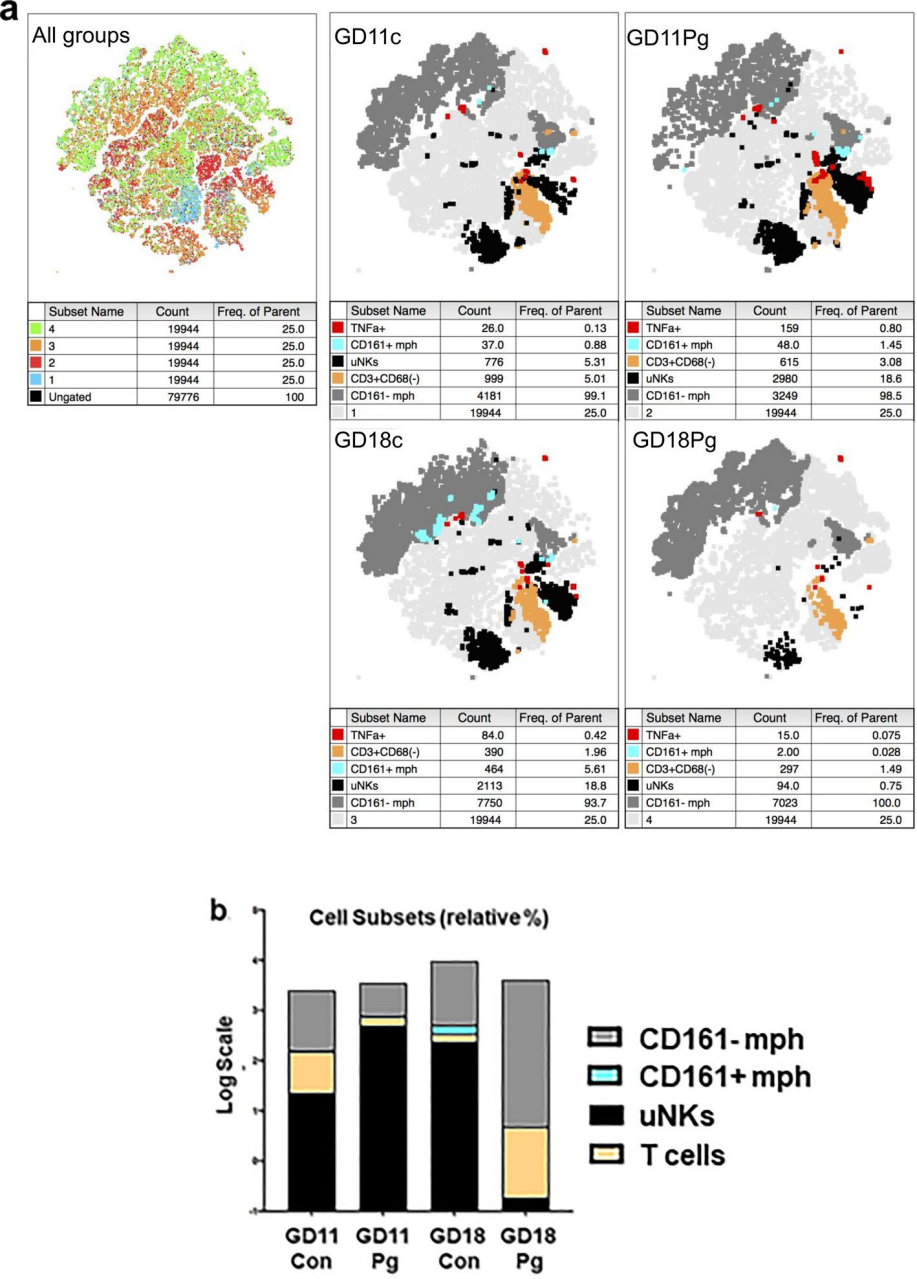
Overview of placental bed leukocyte populations identified by flow cytometry. (**a**) Clustering analysis by tSNE demonstrated population differences free of batch effects and identified corresponding immune cell populations in each treatment group. Processed datasets from all treatments (n = 4 per group) were analyzed with FlowJo’s t-SNE and Flow Means plug-in (FlowJo v10.6.2, Treestar, Ashland, OR). (**b**) Bar chart shows relative proportion of each immune cell subset included in the analysis. Individual populations are shown in supplement file, Fig. S3b.

Processed datasets from all treatment groups and gestational ages were combined and analyzed by t-distributed stochastic neighbor embedding (tSNE), which generated unbiased population cluster maps (Fig. 4a). Unbiased clustering identified a subset of CD161a+ mesometrial MΦs in all groups. However, neither infection nor gestational age significantly altered this subpopulation of MΦ. *P. gingivalis*-infected specimens were found to have significantly more uNK cells at GD11 than control tissue (Fig. 4b, Fig. S3b in Supplement File), with controls having 3.9% uNKs and the *P. gingivalis* group having 14.9% uNKs (P ≤ 0.022) out of total leukocytes. Population means of MΦs, and T cells were not significantly different between groups (Fig. S3b in Supplement File). Production of TNFα by leukocyte populations was also assessed. At GD11, there was no significant difference in the number of TNFα+ cells between control and infected groups (Fig. S3c in Supplement File). At GD18, there was no significant difference in the number of TNFα + MΦ and uNK cells between control and infected groups (Fig. S3c, Supplement File). However, TNFα + T cells were reduced in infected specimens (1.01% in *P. gingivalis* vs 12.56% in controls, (P ≤ value 0.048).

Immunostaining was used to determine the location of uterine MΦ, uNK cells, and T cells in GD11 and/or GD18 uteroplacental specimens. MΦs were double stained for CD161+ and CD68 since CD68+/CD161a + MΦ had not been previously reported. CD68 + cells were detected around spiral arteries. CD161+/CD68+ cells were scant and found to be randomly scattered through the myometrial stroma of all groups (Fig. S4 in Supplement File). None were detected in the decidua.

**Figure 5:**
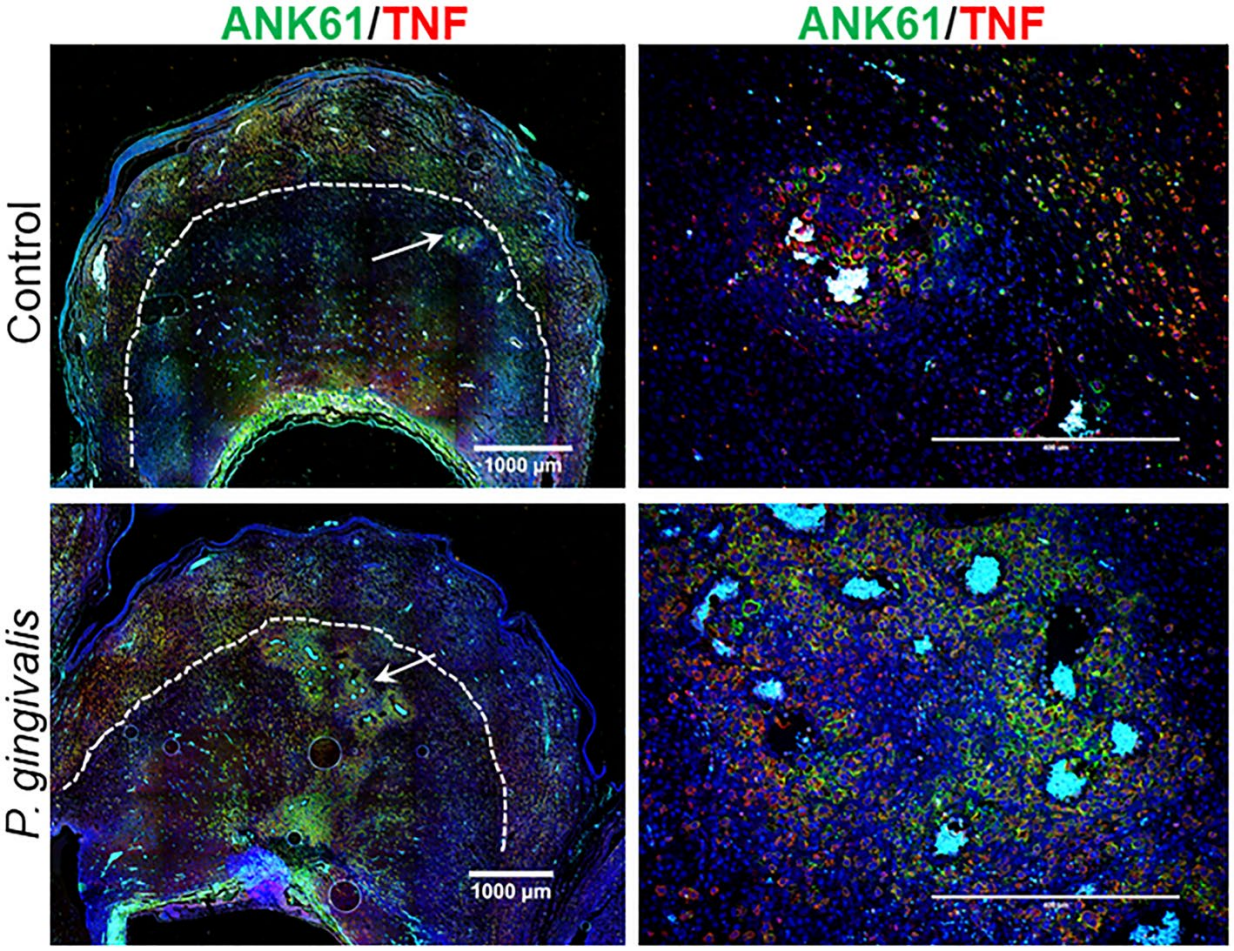
In situ distribution of uNK cells in control and *P. gingivalis*-infected specimens collected at GD11. uNK cells were identified with an antibody to Ank61 (green), some of which are also positive for TNF (red). Left panels are tiled composites of individual pictures taken at × 10 magnification with an EVOS Auto FL imaging system. Scale bar = 1000 µm. Dashed lines demarcate the junction between decidua and myometrium. White arrows mark the spiral artery segments pictured in corresponding magnified images on the right (white scale bar = 200 µm). Representative images of GD18 specimens and isotype controls are found in Supplement File Fig. S5.

UNK cell populations were identified by double staining using the NK marker, a-natural killer cell activation structures (ANK61) combined with TNF or granzyme B (Fig. 5, Fig. S5 in Supplement File). In both GD11 and GD18, uNK cells were concentrated around spiral arteries of control and infected specimens. At GD11, 8 of 14 (57%) of infected specimens had a heavy concentration of uNK cells surrounding decidual spiral arterial segments compared to 2 of 13 (15%) controls (P < 0.05 by Fisher’s exact test). Six of 14 (43%) infected specimens versus 11 of 13 (85%) controls had scant numbers of uNK cells surrounding decidual spiral arterioles. In GD18 specimens, Ank61+/TNF+ cells were primarily located in the outer periphery of the mesometrial triangle (Fig. S5a, Supplement File).

Normally, uNK cells express granzyme B^28^ and form immature activating synapses that are not cytolytic^29^. UNK cells were dual stained with Ank61 and granzyme B to determine if these cells were associated with perivascular necrosis detected in infected GD11 specimens. There was no difference in the proportion of Ank61+/granzyme B+ uNK cells among control and infected groups (79 ± 12% in controls vs. 71 ± 13% in infected, N = 8/group, P = 0.2932 by unpaired t test). Granzyme B positive uNK cells with immature activating synapses were present in both control and infected specimens, but degranulation by these cells was not detected in either group (Fig. S5b in Supplement File).

**Figure 6:**
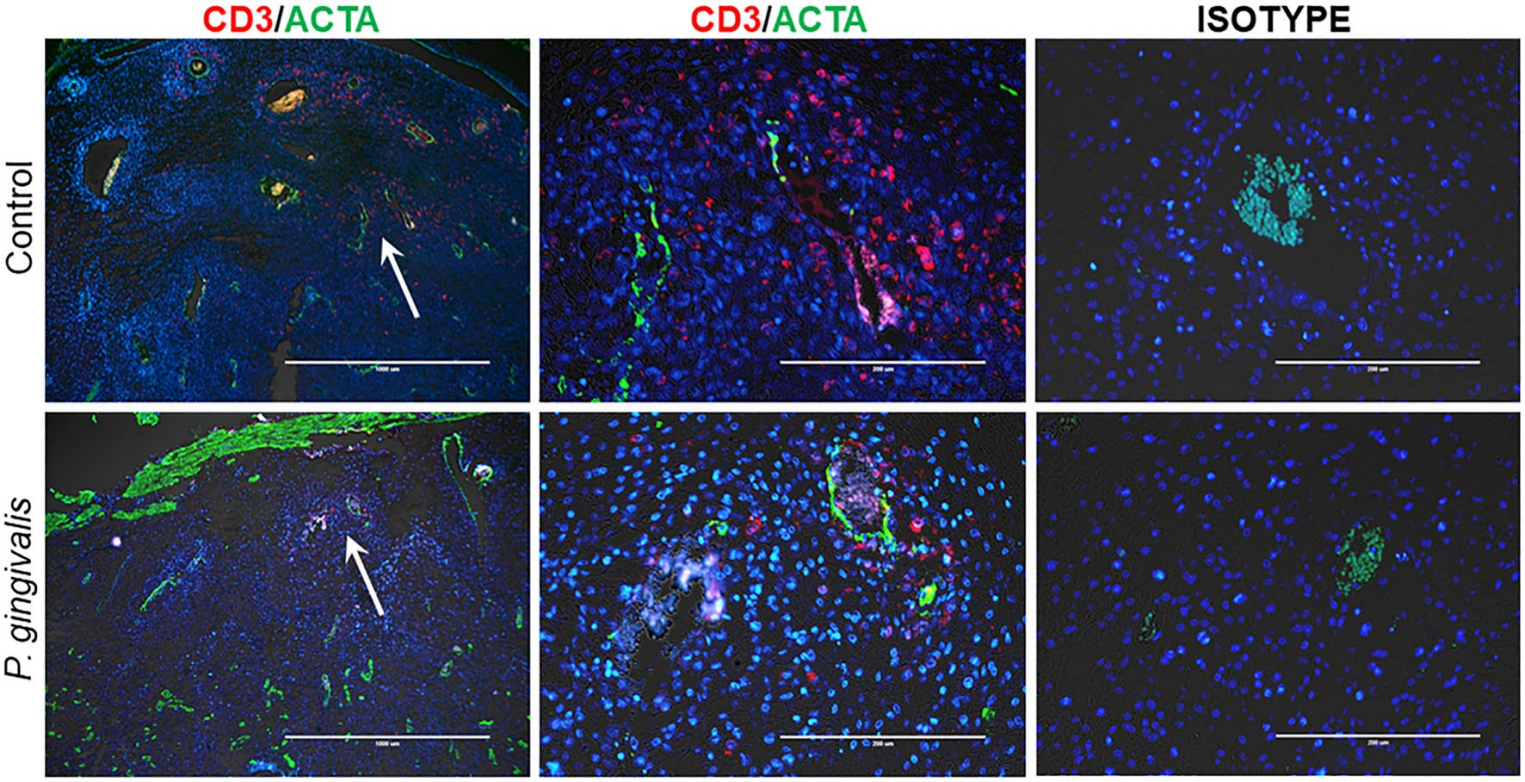
In situ detection of T cells (CD3+, red) and VSMC (ACTA+, green) in GD18 placental bed sections. Left panels are low magnification (× 4) images, scale bar = 1000 µm. White arrows mark the spiral artery segment pictured in the corresponding right panels. White scale bar = 200 µm. All images were captured with an EVOS Auto FL imaging system.

To assess the relationship between T cells and spiral arteries, GD18 specimens were dual stained for CD3 and smooth muscle actin (ACTA) (Fig. 6). CD3+ cells were localized to the outer periphery of the spiral arteries within the outer myometrium in both control and infected specimens.

In summary, *P. gingivalis* infection resulted in an increased number of uNK cells surrounding the spiral arteries during mid-gestation (GD11). By GD18, infected dams had a significant reduction in mesometrial TNFα + T cells found in the deeper myometrium surrounding the spiral arterial segments.

### *P. gingivalis* alters expression of interleukin 18 and high temperature requirement A1 in GD11 placental bed

To assess if infection altered the uterine microenvironment, placental bed specimens were analyzed for the expression of genes implicated in spiral artery remodeling by regulating decidualization, inflammation and oxidative stress, uNK cell dynamics, VSMC dedifferentiation, or angiogenesis^26,30–35^. Placental specimens from GD11 and GD18 groups were examined for the expression of interleukin 1β (*Il1b*), *Il6*, *Il10*, *Il12b, Il15, Il18, tumor necrosis factor (Tnf),* C–C Motif Chemokine Ligand 11(*Ccl11*)*,* transforming growth factor beta 1*(Tgfb1)*, activin subunits: inhibin subunit beta A (*Inhba*) and inhibin A (*Inha*), activin antagonist follistatin-like 3 (*fstl3*), oxidative stress marker high temperature requirement A1 (Htra1), and vascular endothelial growth factor A (*Vegfa*). Gene expression was measured by RT-qPCR with beta-actin *(Actb*) used as the reference gene^36^.

**Figure 7:**
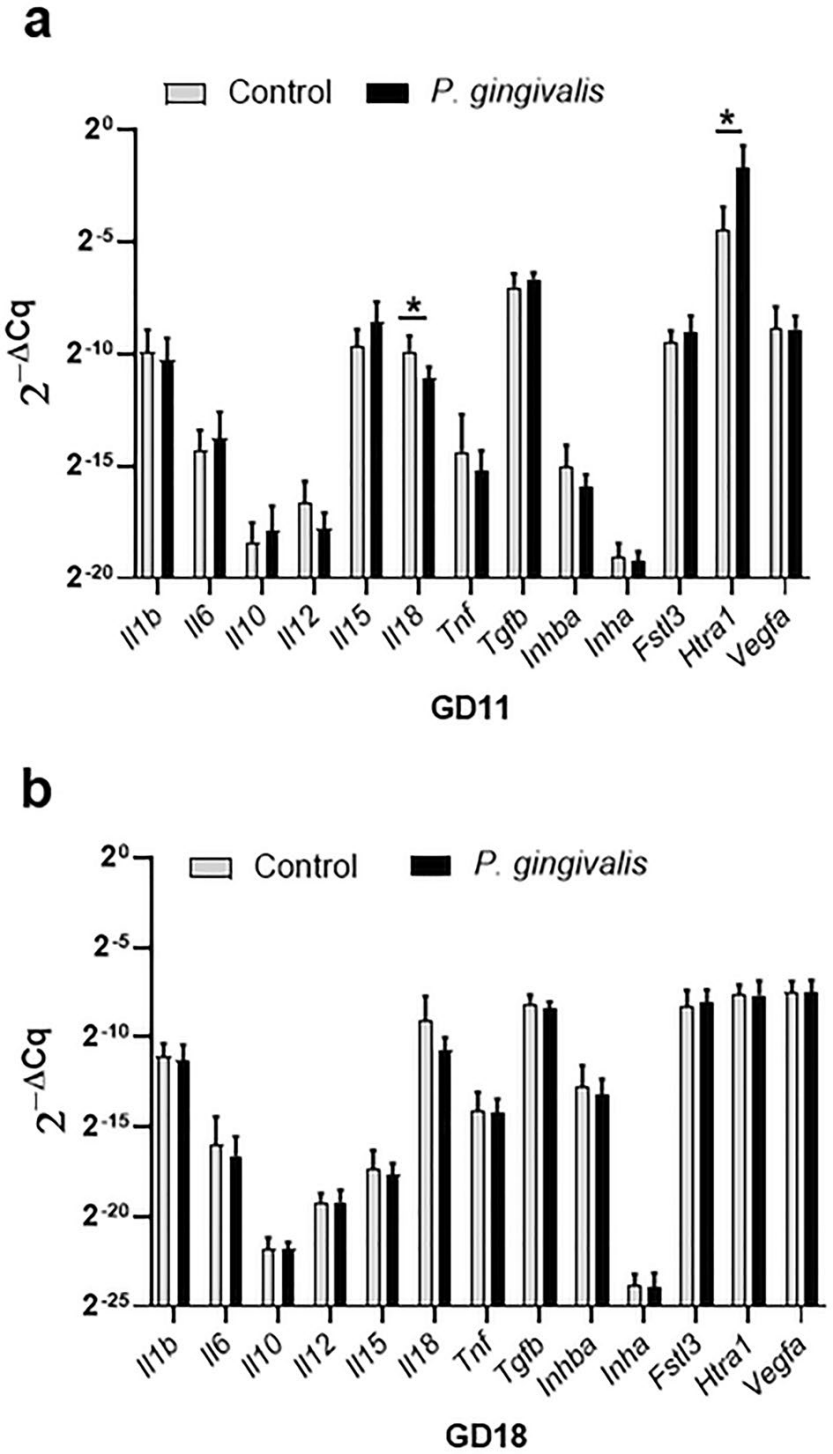
Placental bed gene expression profiles of GD11 (**a**) and GD18 (**b**) specimens. Cytokine and chemokine gene expression measured by RT-qPCR. Values are the mean 2^−ΔCq^ ± SD determined by the comparative Cq method^36^ using *Actb* as the reference gene (n = 10). *Indicates 2^−ΔCq^ values that were significantly different (P < 0.05) by unpaired student’s t test.

At GD11, *P. gingivalis* infected specimens had a reduction in *Il18* expression coupled with an increase in *Htra1* expression (Fig. 7a, P < 0.05). However, by GD18 both *Il18* and *Htra1* expression levels were equivalent between control and infected groups (Fig. 7b). All other gene expression levels were equivalent between gestational age matched control and infected groups.

**Figure 8:**
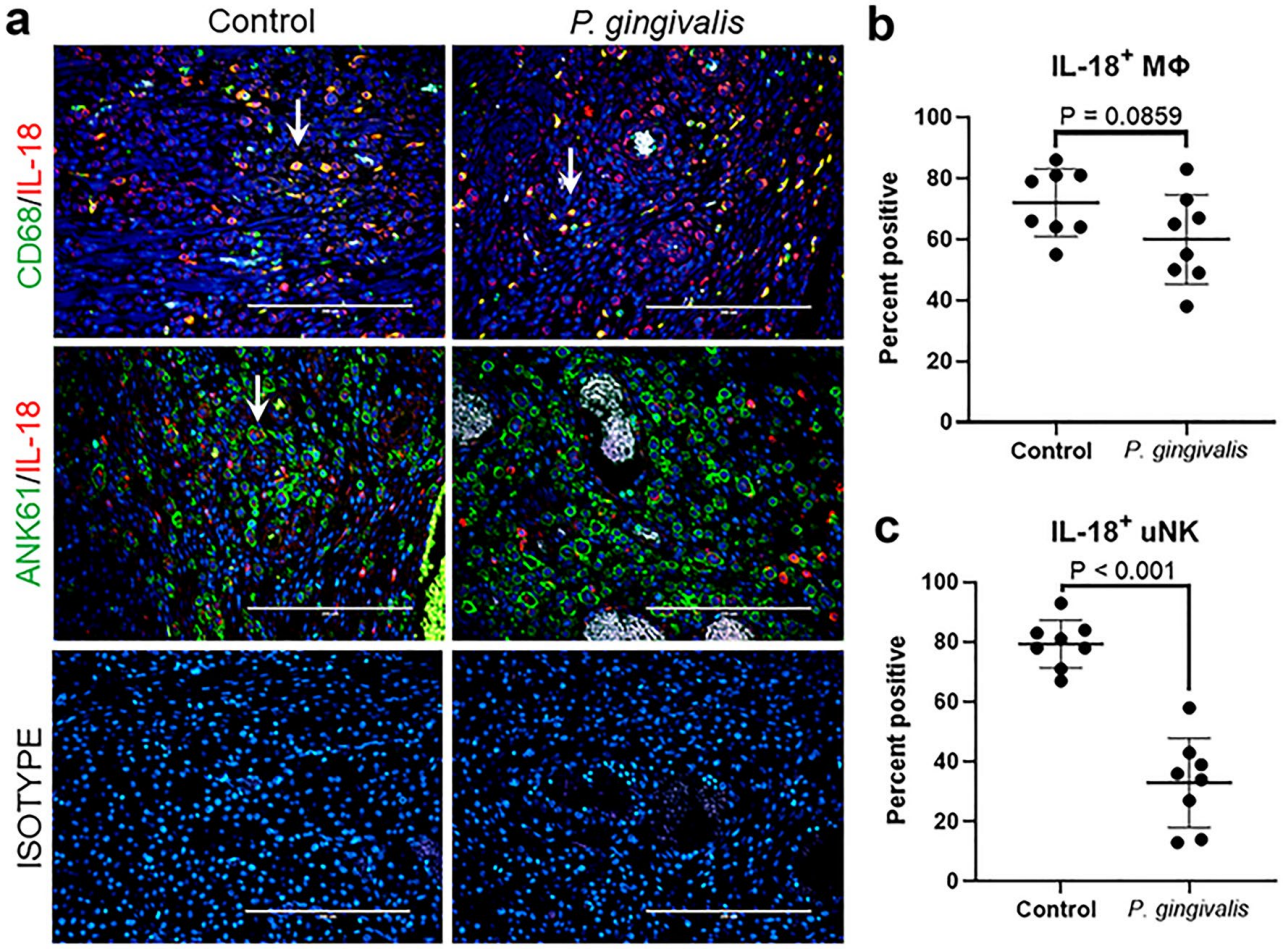
Impact of *P. gingivalis* infection on IL-18 (red) positive MΦ (CD68, green) and uNK cells (Ank61, green) in GD11 specimens. (**a**) Representative images of CD68+/IL-18+ MΦs or Ank61+/IL-18+ uNK cells taken with an EVOS Auto FL imaging system. White arrows indicate double positive cells. Scale bar = 200 µm. (**b**) Percent CD68+/IL-18+ MΦs. (**c**) Percent Ank61+/IL-18+ uNK cells. Horizontal lines in each graph indicate the mean ± SD. Data was analyzed by unpaired student’s t test.

Immunofluorescent staining of GD11 placental bed specimens was used to assess changes IL-18 and HTRA1 at the protein level. For IL-18 analysis, tissues were dual stained with MΦ marker CD68 or uNK marker Ank61 (Fig. 8a). IL-18 staining was strongest in myometrial MΦs in both control and infected groups. The proportion of IL-18 positive MΦs was equivalent in both control and infected groups (P = 0.0859, Fig. 8b). However, the proportion of IL-18 positive uNK cells was reduced in infected specimens (P < 0.001, Fig. 8c).

Immunostaining for HTRA1 was done in conjunction with trophoblast marker, cytokeratin 7 (CYTO7), or smooth muscle cell marker, ACTA (Fig. 3c). CYTO7 was chosen because trophoblasts are known to express HTRA1^37^. ACTA was selected since HTRA1 is essential for VSMC differentiation into the contractile phenotype^38,39^. Overall, the distribution of HTRA1 staining was different between control and infected specimens. Stromal cells in both groups were positive for HTRA1, but staining was more intense and widespread in infected specimens (Fig. 3c). Endovascular invasive trophoblasts were positive for HTRA1, but staining was more intense in controls than in infected tissues (Fig. S3). There was no observable difference between HTRA1 expression in spiral VSMC from control and infected groups. Collectively, infection-induced changes in placental bed IL-18 and Htra1 involved cells that participate in spiral artery remodeling (i.e., uNK cells and endovascular invasive trophoblasts).

## Discussion

Impaired spiral artery remodeling underlies a range of obstetrical disorders including preterm delivery and preeclampsia with or without fetal growth restriction^1^. We previously demonstrated that experimental infection with *P. gingivalis* disrupts the physiologic remodeling of the spiral arteries^10,11^, which could explain how *P. gingivalis* promotes a range of seemingly disparate obstetrical syndromes^4–8,40^. One limitation of our previous work is that it could not establish if *P. gingivalis-*mediated disruption of spiral artery remodeling occurs prior to invasion by extravillous trophoblasts. Herein we show that *P. gingivalis*-mediated disruption of spiral artery remodeling is evident by GD11, which includes alterations in placental bed Htra1 and IL-18, uNK cell populations as well as reduced endovascular trophoblast invasion. Taken together, these results suggest *P. gingivalis* disrupts both trophoblast-independent and dependent phases of spiral artery remodeling. Given that *P. gingivalis* can be detected in the placental bed stroma in this study as well as in our previous work^10,11^, we propose that *P. gingivalis* alters the uterine microenvironment, which in-turn disrupts the coordinated regulation of spiral artery remodeling.

Htra1 is essential for placentation and optimal spiral artery remodeling^41^. Moreover, disturbances in Htra1 regulation and function are implicated in preeclampsia, and FGR^32,42–45^. Mechanistically, Htra1 overexpression may have a negative impact on both trophoblast-independent and trophoblast-dependent phases of spiral artery remodeling. For example, secreted Htra1 antagonizes TGF-β and activin-mediated signaling^46^, which is important in uNK cell-mediated remodeling of spiral arteries^26^. Ectopic overexpression of Htra1 inhibits human extravillous trophoblast migration in vitro^32,47^. Thus, decidual and myometrial overexpression of Htra1 detected in GD11 infected specimens, especially within the perivascular stroma, may be contributing to IRSA.

At least in rodents, IL-18 is important for successful pregnancy and optimal spiral artery remodeling^48^. In the mouse, uNK cells become the principal source of IL-18 production by GD7-8, which may act as autocrine signaling mechanism to maintain uNK cell activation necessary for spiral artery remodeling^31^. Moreover, ablation of IL-18 in mice reduces the extent of spiral artery remodeling by as much as 30 to 44%^48^. In our model, *P. gingivalis* infection resulted in a significant reduction of IL-18+ uNK cells at GD11 that coincided with a significant reduction in spiral arterial lumen size combined with increased retention of spiral VSMC. While our results do not establish a causal relationship between uNK cell changes and IRSA, they provide a compelling argument for pursuing further studies in the rat since in this species endovascular invasive trophoblasts reach the inner myometrial segments of the spiral arteries.

*Porphyromonas gingivalis* utilizes various virulence strategies to manipulate host defenses that may be relevant to spiral artery remodeling. For example, *P. gingivalis* invasion of host cells suppresses their production of cytokines/chemokines that recruit leukocytes to the infected site^49^. Proteases secreted by *P. gingivalis* also degrade cytokines in the local microenvironment^50^. These elements may be contributing to the lack of inflammatory cytokine/chemokine expression in the decidua, the lack of cytotoxic uNK cells, and the decrease in IL-18+ uNK cells in infected specimens.

We did not expect to find endovascular invasive trophoblasts in GD11 specimens since previous studies reported that invasion by endovascular invasive trophoblasts in rats begins around GD13 or 14^19^. This may be due to our system of gestational staging in which we designate the day of breeding as GD0 whereas others designate the breeding date as GD1^19^. Regardless, this study showed a significant reduction in invasive trophoblast invasion in *P. gingivalis* infected specimens that persisted in later stages of pregnancy^10,11^. The increased density of uNK cells in infected specimens may be one contributing factor since uNK cells restrain endovascular trophoblast cell invasion in the rat^51^. It is also possible that *P. gingivalis* directly affected endovascular invasive trophoblast migration and/or viability. Based on in vitro studies, clarified supernatant from *P. gingivalis* cultures inhibits HTR-8/SVneo migration, which is an immortalized extravillous trophoblast cell line^52^. Notably, *P. gingivalis* packages and secretes various virulence factors in outer membrane vesicles that can diffuse through tissue or the bloodstream and enter host cells^53^. Given the location of *P. gingivalis* within the decidua, it is possible that virulence factors secreted into the surrounding tissue microenvironment or bloodstream, reach endovascular invasive trophoblasts inhibiting their migration. Invasion of HTR-8/SVneo by *P. gingivalis* can also affect their viability by inducing G1 arrest and apoptosis^54,55^. We did not observe *P. gingivalis* in association with endovascular invasive trophoblasts in any of our stained specimens, so it is unclear if this phenomenon would be relevant in our model.

An unexpected finding was the significant decrease in CD3+/TNF+ cells in the GD18 infected group. In situ staining located these cells in the outer periphery of the spiral arteries. The biological significance of this is unknown. However, it may be relevant to spiral artery remodeling and merits further investigation since Veerbeek et al. reported a reduction in CD3+ cells in placental bed biopsies from women with IRSA and preeclampsia^56^.

To replicate a natural chronic infection, female rats receive a finite number of oral inoculations before breeding, which establishes generalized periodontitis and oral dysbiosis that persists through pregnancy^11,57^. In our system, rat breeding begins 1 week after the last oral inoculation creating a 2 to 6 weeks gap between the last oral inoculation and pregnancy. Therefore, intrauterine infection is caused by an endogenous source of *P. gingivalis* that is adapted to the rat host. This in part may explain why infected dams do not exhibit systemic inflammation defined as increased circulating cytokine/chemokine concentrations.

Pregnancies complicated by IRSA have a greater risk for maternal and infant morbidity and mortality^58^. IRSA was initially thought to be a specific complication of early-onset preeclampsia with and without fetal growth restriction, but it is now apparent that IRSA also underlies early pregnancy loss, preterm labor, preterm premature rupture of membranes, late spontaneous abortion, and abruptio placentae^1–3^. Of these conditions, intrauterine infection with *P. gingivalis* is linked to early recurrent miscarriage, preterm labor, preterm premature rupture of membranes, and preeclampsia with or without fetal growth restriction^4–8^. Although epidemiologic studies concerning *P. gingivalis* infections during pregnancy are few, colonization rates in complicated pregnancies range between 16 and 92% vs 2 and 70% in normal pregnancies^5–9^. Our rat model of infection offers a probable mechanism (i.e., IRSA) whereby *P. gingivalis* could promote such a diverse spectrum of pregnancy complications. The changes that we observed in the placental bed of infected animals suggests *P. gingivalis* disrupts both trophoblast-independent and trophoblast-dependent stages of spiral artery remodeling. This study also highlights the potential value of dysregulated IL-18, Htra1, uNK cells, and TNF + T cells in the pathogenesis of *P. gingivalis*-induced IRSA.

## Methods

### Handling and infection of rats

All experimental protocols were approved by the University of Wisconsin Institutional Animal Care and Use Committee (#V005576). All methods were conducted in accordance with relevant guidelines and regulations set forth by University of Wisconsin Institutional Animal Care and Use Committee and OLAW of the National Institutes of Health. All experimental procedures and data reporting are compliant with ARRIVE guidelines (www.arriveguidelines.org). At the start of the study, 6 to 8 weeks old specific pathogen free Sprague Dawley (SD) and Wistar (WIS) female rats were obtained from Charles River International Laboratories, Inc., Kingston, NY. Animals were housed under biosafety level 2 housing within the same room and maintained under 12-h light cycles and fed sterile food and water adlib. SD and WIS rats were comingled beginning 2 weeks before inoculation and throughout the study so that any differences that may be present in their microbiome could be equalized between both rat strains. Animals were weighed before inoculation, after the end of the inoculation period and at time of necropsy. No differences were observed in the rate of weight gain among control and infected groups. GD11 dam weights at time of necropsy were 341 ± 72 in controls and 343 ± 42 in infected. GD18 dam weights were previously reported^11^. Control animals were always handled before infected animals to prevent cross contamination.

Oral inoculations were performed as already described^11^. Animals received sterile 2% carboxymethylcellulose (CMC) or 1 × 10^9^ CFU of *P. gingivalis* strain A7UF suspended in 2% CMC administered orally for 4 consecutive days, on alternating weeks over a 12-week period. The CFU of each inoculate was confirmed by culture.

For breeding, one female rat was placed with a male of the same strain overnight. Breeding was confirmed the next morning by the presence of sperm within vaginal lavage fluid. The day of breeding was deemed gestation day (GD) 0. Pregnancy was confirmed by monitoring daily weight gain and by abdominal palpation at GD 10 or 11. Dams underwent no more than two breeding cycles to be included in the study.

For purposes of rigor, multiple smaller experiments were conducted, which included 5 to 6 dams per strain per treatment group (control and infected).

### Cultivation of *P. gingivalis* and preparation of oral inoculates

Culture methods and preparation of inoculates was performed as previously described^11^.

### Necropsy and tissue processing

Pregnant dams were euthanized and necropsied at GD11 or GD18. Blood was collected for cytokine analysis, processed and analyzed as previously described^11^. Utero-placental units were randomly assigned to histologic evaluation or gene expression analysis. Excess uterine tissue was trimmed from the mesometrial triangle of tissues selected for histology and fixed in 10% buffered formalin overnight then washed in distilled water and transferred to 70% ethanol until processing. Placental bed specimens selected for gene expression analysis were separated from the placenta and fetus, and excess uterine tissue was removed before immersion in Trizol. Tissues were flash frozen in liquid nitrogen and stored at – 80 °C until processing.

### Histology, immunofluorescent staining, and morphometry of GD11 specimens

H and E stained uteroplacental specimens were coded so that the interpreter was blinded to treatment. Utero-placental specimens were evaluated for fibrinoid deposition, infarcts/thrombosis, hemorrhage, and coagulative necrosis. Immunofluorescent staining was performed as previously described^10,11^. Specimens to be stained with anti-CYTO7 antibody underwent antigen retrieval^59^ with Tris–EDTA buffer pH 9 [10 mM Tris base with 0.05% EDTA], all other specimens were treated with citrate buffer pH 6 [10 mM sodium citrate with 0.05% Tween 20]. Tissues were incubated in either buffer 95 °C for 5 to 10 min. Antibodies used in this study are summarized in Supplement File, Table S1.

Stained tissue sections were observed and imaged using an EVOS AutoFL microscope system (Life Technologies, Grand Island, NY) as previously described^11^. For imaging purposes, camera settings were optimized using the tissue section with the highest positive fluorescent signal, which were kept the same during imaging all other tissue sections within the same staining experiment.

Morphometric analysis for spiral artery remodeling was performed as previously described^11^. Calibrated images of each mesometrial triangle were analyzed with ImageJ software 1.52v (Rasband, National Institutes of Health, USA, https://imagej.nih.gov/ij/download/html).

### Maternal serum chemokine/cytokine analysis

Serum collected at time of necropsy was stored at − 80 °C until analysed. Samples from all gestation (GD11 and GD18) and treatment (control and infected) groups were analysed at the same time within the same assay. Briefly, 50 µL of serum were analyzed for IL-1α, G-CSF, IL-10, IL-17A, IL-1β, IL-6, TNFα, IL-4, GM-CSF, IFNγ, IL-2, IL-5, IL-13, IL-12p70, Eotaxin, GRO-α, IP-10, MCP-1, MCP-3, MIP-1α, MIP-2, and Rantes (Rat Cytokine & Chemokine 22-plex ProcartaPlex Panel, Invitrogen, Catalog #EPX220-30122-901) as per the manufacturer’s instructions. Samples were analysed with a Luminex 200 instrument (Thermo Fisher Scientific, Waltham MA).

### Flow cytometry experiments

Six to 8 implantation sites from each dam were randomly selected and pooled for flow cytometry experiments. Fetus, placenta, and excess uterine tissue was removed from each metrial triangle prior to cell isolation. Cell isolations were performed as described^59^ with the following modifications. Mechanical tissue dissociation was performed with a GentleMACS Tissue Dissociator (Miltenyi Biotech.com) using the spleen setting. Leukocyte enrichment by centrifugation was achieved with a 70%/40%/20% Percoll (SigmaAldrich.com) gradient at 500×*g* for 30 min at 4 °C. Cells were collected from the middle interface and passed through a 70 µm strainer and washed in PBS before staining.

Antibodies and reagents used for flow cytometry are summarized in Table S2 in supplement file. Fluorescence minus one controls were included for each antibody and each experiment. Staining was performed according to manufacturer’s instructions. Outer surface receptors were stained first, fixed with 4% paraformaldehyde for 30 min, washed, and stored overnight at 4 °C prior to permeabilization and staining for intracellular markers (day 2). Stained cells were analyzed with a BD LSRFortessa Cell Analyzer (www.BDbiosciences.com) within 72 h of staining. Each flow cytometry experiment contained 2 or more groups. Compensation was determined using single-stained beads and unstained cells. Positive versus negative parameters for each channel were refined using unstained cells and cells stained with fluorochrome combinations to identify spectral overlap fluorescence minus one (FMO) controls. Gating strategy is shown in Supplement Fig. S3a.

Analysis of raw flow cytometry data was initially performed with FlowJo v10.6.2 (Flow Jo, LLC, Ashland, OR). Live, single, CD45+ leukocytes from each specimen were down-sampled to an equal number and then concatenated into their respective treatment groups based on gestational age and infection status (N = 4 per treatment group). To avoid bias, tSNE was performed “blinded” to marker identity. For verification purposes, results were then manually gated (Supplement File, Fig. S3a). Populations of interest were uterine Natural Killer (uNK) cells (defined as CD3−CD68−CD161+), T cells (CD3+CD68−), and macrophages (CD68+CD3−). These populations were identified both through clustering and manually with FlowJo’s t-SNE and Flow Means plug-in.

### Gene expression analysis

At time of necropsy, the mesometrial triangle and decidua was removed and excess uterine tissue was trimmed away from the mesometrial triangle before immersion in Trizol Reagent (Life Technologies, Cat# 15596-018) at a ratio of 1 mL of reagent per 50–100 mg of tissue. Samples were stored at − 80 °C in RNAase free tubes until processing. RNA extraction and assessment of RNA quality was performed as previously described^11^. Total RNA was converted to cDNA and qPCR was performed with GoTaq 2-Step RT-qPCR System (Promega Corp, catalog #A6010, Madison, WI) using primers listed in Table S2 according to manufacturer’s instructions. RT-qPCR were performed as previously described^11^. All primer pairs listed in Table S3 have similar primer efficiencies determined by logarithmic PCR amplification plots^11,36^. RT-qPCR reactions were performed with a LightCycler 96 Roche Real-Time PCR system (Roche Diagnostics, Indianapolis, IN) using the same amplification conditions as already described^11^.

### Statistical analysis

One way ANOVA, student’s t test, and Fisher’s exact test was performed with GraphPad Prism version 8.4.2 Software (GraphPad Software, LLC), with P < 0.05 being considered significantly different.

## Supplementary Information

Supplementary Information.

## Data Availability

Raw data were generated at University of Wisconsin-Madison School of Veterinary Medicine. The data that support the findings of this study are available from the corresponding author upon reasonable request.
