# Supplementary material for: Porphyromonas gingivalis-mediated disruption in spiral artery remodeling is associated with altered uterine NK cell populations and dysregulated IL-18 and Htra1
Source: Sci Rep. 2022 Aug 30;12:14799. doi: 10.1038/s41598-022-19239-9 (PMC9427787; doi:10.1038/s41598-022-19239-9)
Supplement: Supplementary file 1 — Supplementary Information. [file 41598_2022_19239_MOESM1_ESM.pdf]

Porphyromonas gingivalis-mediated disruption in spiral artery remodeling is associated with altered uterine NK cell populations and dysregulated IL-18 and Htra1

Tanvi Tavarna, Bryce Wolfe, Xiao-jun Wu, and Leticia Reyes*

Department of Pathobiological Sciences, University of Wisconsin - Madison, School of Veterinary Medicine, Madison, WI. USA

Materials

Table S1: Antibodies used for immunostaining

Target antigen	Dilution	Host Organism	Catalog #, Manufacturer
P. gingivalis	1:2000	Rabbit polyclonal	C7947, Custom made [5]
Cytokeratin-7	1:200	Mouse (clone LP1K)	Ab20206, Abcam (Cambridge, MA)
Smooth muscle actin	1:200	Mouse (clone 1A4-asm-1)	MA5-11547, Invitrogen (Rockford, IL)
Htra1	1:200	Rabbit polyclonal	PA5-79419, Thermofisher (Waltham MA)
TNF-α	1:200	Rabbit polyclonal	AAR33, Biorad (Hercules, CA)
Ank61	1:200	Mouse	ab36392, Abcam (Boston, MA)
CD3	1:100	Rabbit [Sp7]	ab16669, Abcam (Boston, MA)
Granzyme B	1:100	Rabbit polyclonal	NB100-684, Novus Biologicals (Centennial, CO)
IL-18	1:200	Rabbit monoclonal	Ab223293, Abcam (Boston, CA)
Rabbit Ig ALEXA 594	1:1000	Goat	A-11037, Life Technologies (Grand Island, NY)
Mouse Ig ALEXA 594	1:1000	Goat	A-11032, Life Technologies (Grand Island, NY)
Rabbit Ig ALEXA 647	1:1000	Goat	A32733, Life Technologies (Grand Island, NY)
Mouse Ig ALEXA 647	1:1000	Goat	A32728, Life Technologies (Grand Island, NY)
Rabbit Ig ALEXA 488	1:1000	Goat	A32731, Life Technologies (Grand Island, NY)
Mouse Ig ALEXA 488	1:1000	Goat	A32723, Life Technologies (Grand Island, NY)

Table S2: Antibodies/reagents used for flow cytometry

Target antigen	Catalog #	Manufacturer
CD161a (BUV395)	744055	BD Biosciences
CD3 (BV605)	563949	BD Biosciences
CD45 (ALEXA-647)	202212	Biolegend
CD68 (ALEXA-700)	NB600-985AF700	Novus Biological
TNF-α (biotin)	NBP2-34419B (biotin)	Novus Biological
Isotype (BUV395)	564059	BD Biosciences
Isotype (BV605)	563517	BD Biosciences
Isotype (ALEXA-647)	400130	Biolegend
Isotype (ALEXA-700)	IC002N	Novus Biological
Isotype (biotin)	BP2-62224	Novus Biological
Ghost Dye™ Viability dye (780)	13-0865-T100	Tonbo Biosciences
Cytofix/Cytoperm™ kit	554714	BD Biosciences
Brilliant Stain Buffer	563794	BD Biosciences

Table S3: Primers used for RT-QPCR

Gene	Manufacturer Catalog#
IL1b	Qiagen QT00181657
IL6	Qiagen QT00182896
IL10	Qiagen QT00177618
IL12b	Qiagen QT00188839
IL15	Qiagen QT01813637
IL18	Qiagen QT00183071
Tnf	Qiagen QT00178717
Tgfb1	Qiagen QT00187796
Inhba	Qiagen QT00183918
Inha	Qiagen QT00370258
Fstl3	Qiagen QT00191044
Htra 1	Qiagen QT00178017
Vegfa	Qiagen QT00198954
Actb	Qiagen QT00193473

Supplementary Figures

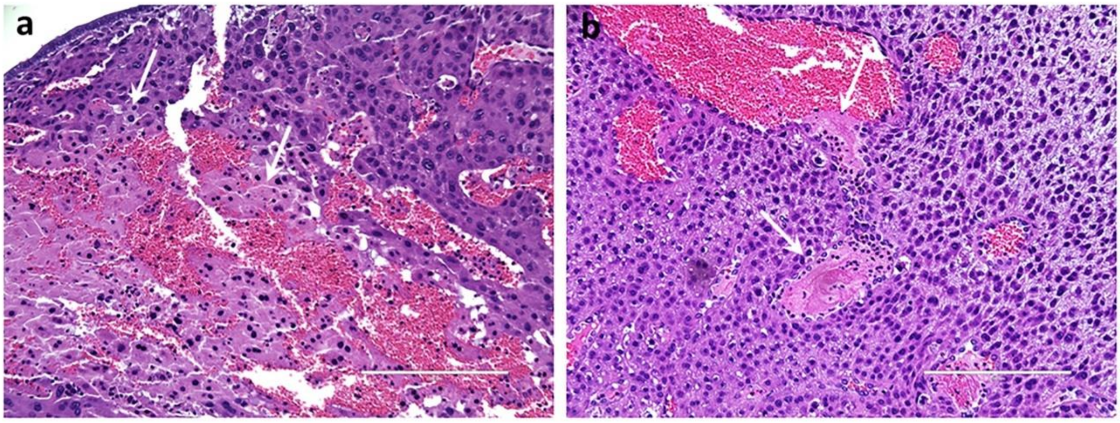

Fig S1 Representative images of GD11 placental bed lesions. **a.** Coagulative necrosis at the placental-decidual junction (white arrows demarcate lesion edge) and **b.** Thrombosis of decidual blood vessels (white arrows). Scale bar = 200 µm.

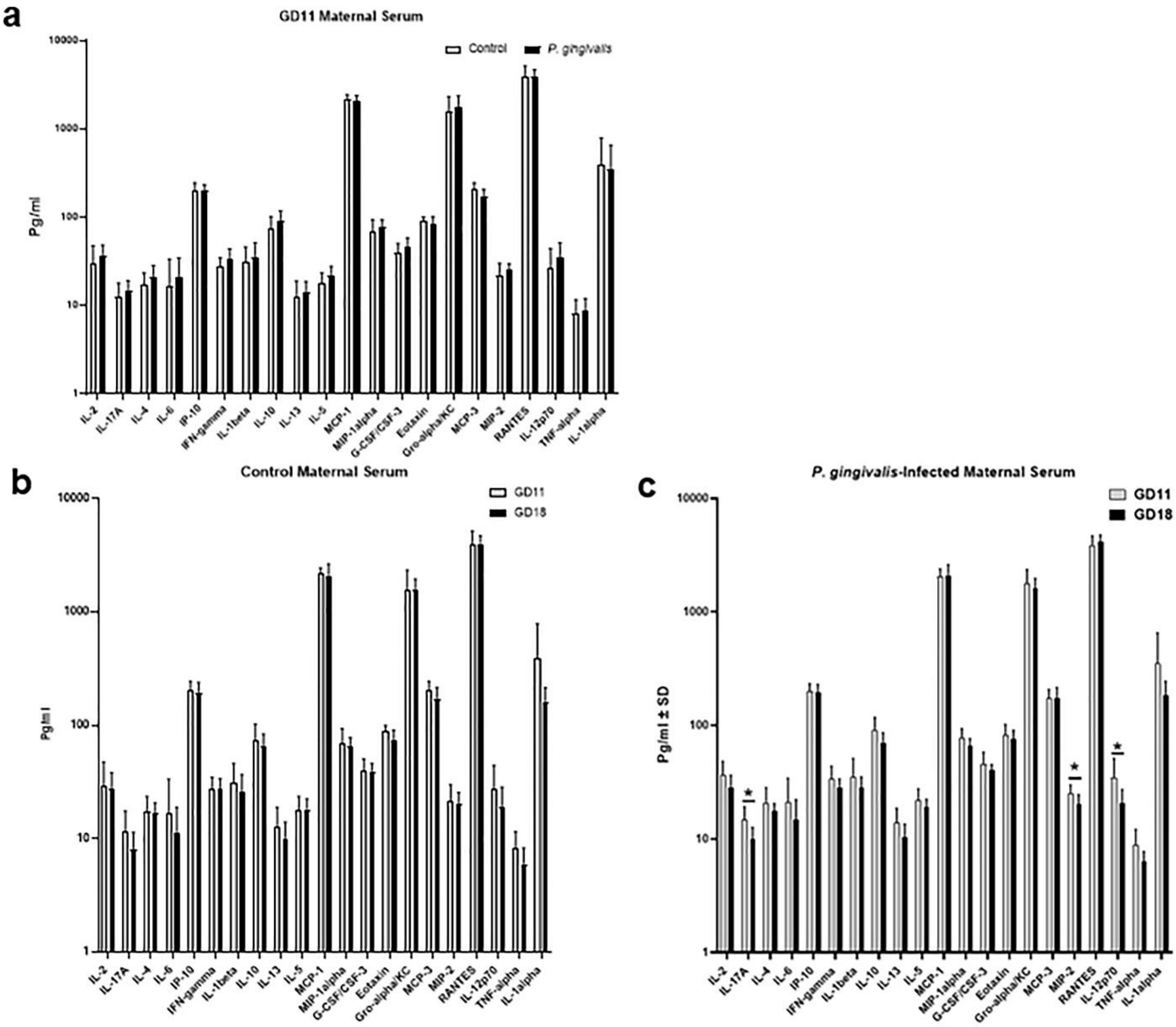

Fig S2. Maternal serum chemokine/cytokine profiles collected at GD11 or GD18. Data represents the mean Pg/ml ± SD (n = 10). Serum samples were analysed with a Cytokine & Chemokine 22-Plex Rat ProcartaPlex™ Panel (Invitrogen, catalog # EPX220-30122-90) using a Luminex 200 System (Thermofisher Scientific). *Indicate groups that are different by unpaired t test ($P < 0.05$).

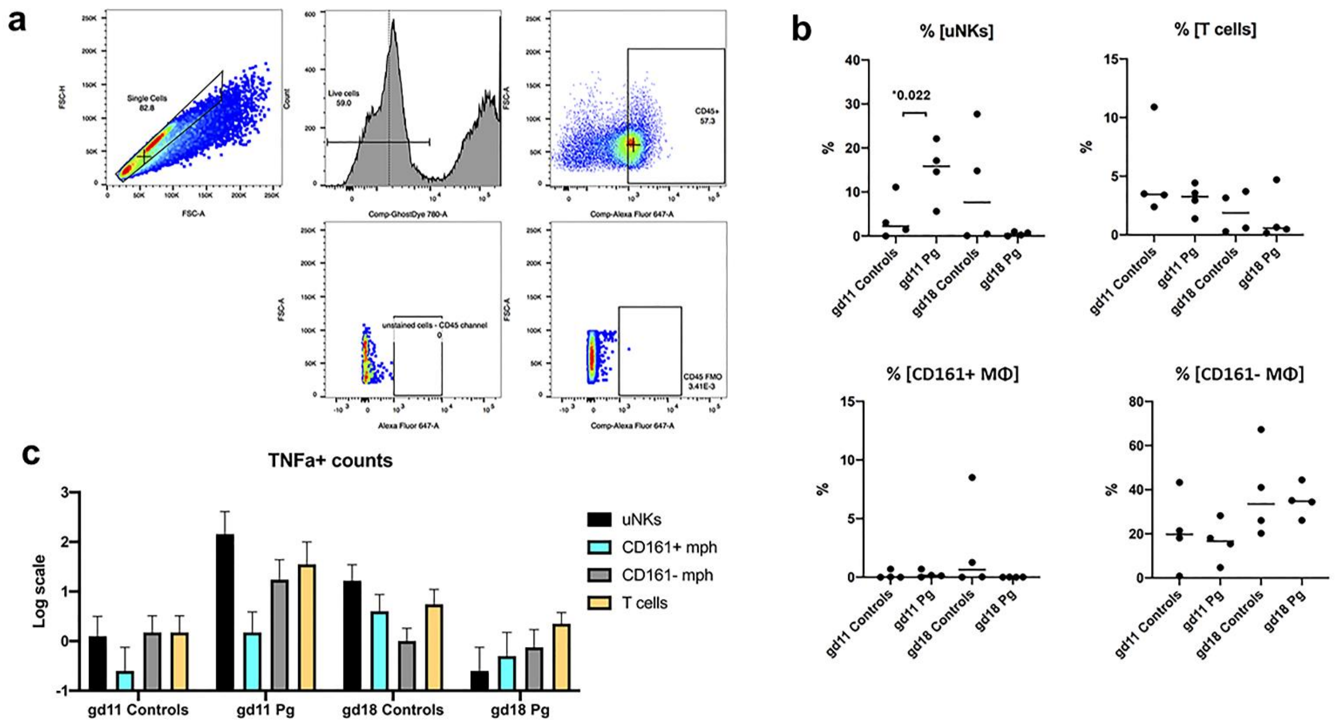

Fig S3. Flow cytometry methods and additional data. **a.** Gating strategy for flow cytometry data analysis. Live, single CD45+ cells were assessed. Unstained cells and fluorescence minus one (FMO) controls were included in each experiment to determine gating parameters. **b.** Percent frequency of immune cell subtypes GD11 and GD18 control and infected groups. GD11 uNK cells were significantly increased in *P. gingivalis* group compared to controls, by Student's t-test. **c.** The number of TNFa positive cells in each subset. GD18 T cells in the *P. gingivalis*-infected group expressed significantly less TNFa compared to controls.

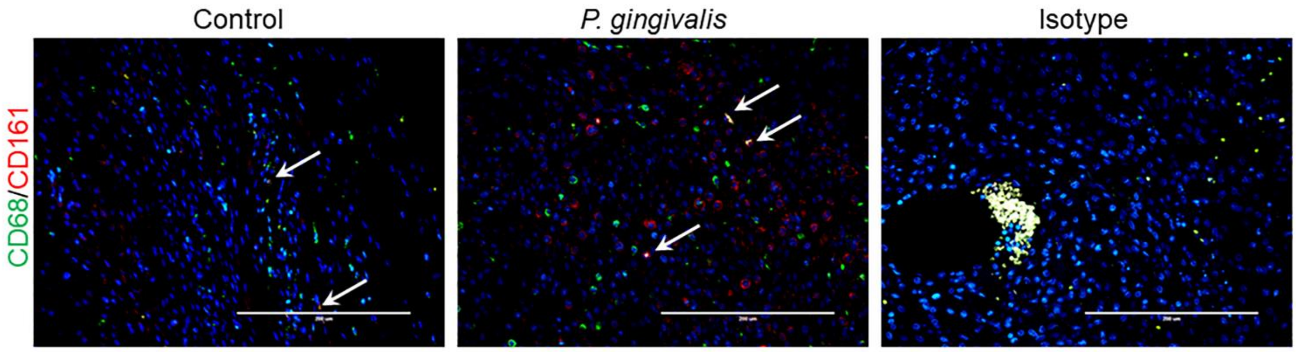

Fig S4. *In situ* location of placental bed CD68+/CD161+ MΦ in control and *P. gingivalis*-infected specimens. White arrows indicate double positive cells. Images were captured with an EVOS AutoFL system. Scale bars = 200 µm.

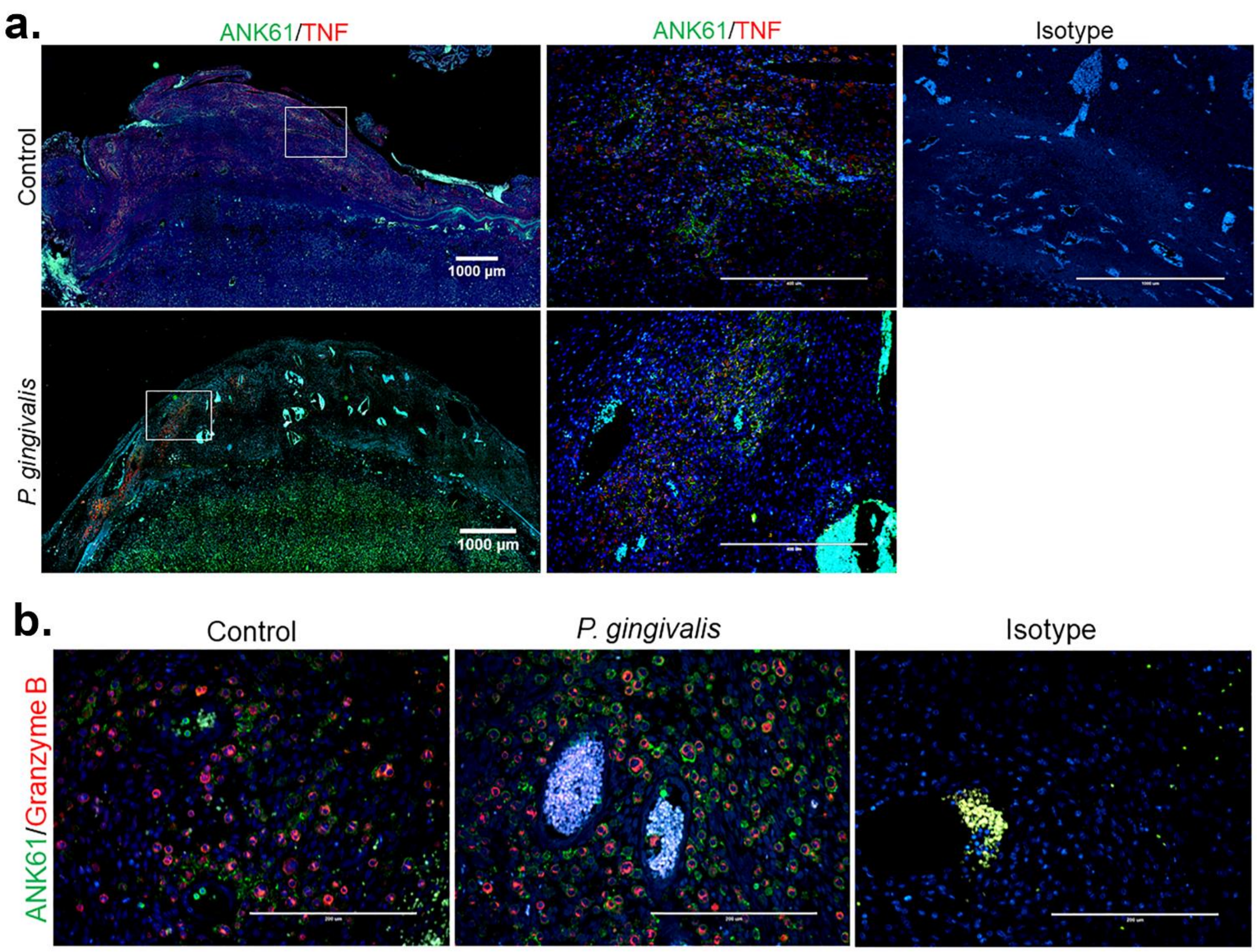

Fig S5. *In situ* detection of uNK cells in GD18 (**a**) and GD11 (**b**) placental bed specimens. **a.** Representative images of GD18 Ank61+ (green)/TNF+ (red) uNK cells within the mesometrial triangle of control and infected dams. Left panels are tiled composites of pictures taken at 10X magnification with an EVOS Auto FL imaging system. Scale bar = 1000 µm. White box indicates location of the corresponding magnified area (right panel). **b**. Representative images of Granzyme B+ uNK cells surrounding the spiral arteries in GD11 control and infected specimens. Scale bar = 100 µm.
